# The Trials of the Profession

**Published:** 1881-11

**Authors:** 


### THE TRIALS OF THE PROFESSION.

BY A CONFIRMED DYSPEPTIC.

Some one has said that it is'nt work that kills, it is worry. This reflection must have owed its origin to some one's observation of a dentist's daily experience. Among the minor trials which tend to hasten a dentist's advent into the insane asylum, (to mention only the lesser evil which may overtake him,) is the having to answer the same old questions a score of times daily. It often happens after the patient has vacated the chair, and while the dentist, exhausted and exasperated by the difficulties of a prolonged operation, is anxious only for the instant to arrive when his patient will say good morning, and leave him at liberty to go out into the back hall and give suitable expression to the emotions inspired by his late experience, that he is required to wait a while and answer a lot of questions such as follow: (I take the liberty of abbreviating such as must be familiar to every dentist.)

Question. "Doctor * * * * * dentifrice?"

Answer. "We make a powder after a formula of our own, which is warranted to remove tartar and arrest decay. You may judge of its efficacy when I inform you that our rivals complain that its introduction is likely to ruin the business and reduce our profession to the condition of a superfluity."

[My partner, Dr. Prostheticus usually chimes in at this point, and advises the questioner to lay in an ample supply of our powder for self and posterity, assuring him that we are about to discontinue its manufacture, owing to the fact of its having been hinted to us that in the probable event of the separation of operative and mechanical dentistry, we would be liable to be ruled out of the chosen fold if it should become known that we employed our leisure in the mechanical performance of preparing tooth powder.]

Question: "I've ruined my teeth * * * * creosote. Don't you think so?"

Answer: "(Oh the dev-eh-eh.) No!"

Question: " * * * * * * * ?"

Answer: "Why its good enough, so's you don't use too much of it."

Question: "What make * * * * ache, if * nerve * dead?"

[To this question I return a variety of answers, such as; the periodontium, as a result of a hyperæsthetic condition of the investing tissues becomes affected, mephitic gases are generated, which passing through the apical foramen, etc., etc., *ad libitum.* This dose, if promptly and rapidly administered, will often throw a man of robust habit into a profuse prespiration, and shut him up at once.]

I will say right here, that I, being of a patient, amiable disposition, suffer a prolonged infliction of this kind of catechizing more frequently than does my partner, Dr. Prostheticus, for he, although always respectful and forbearing, unconsciously assumes an expression of countenance so profane about the time the fourth question is put, that the patient usually retreats precipitately down stairs.

Question: "Is Doctor * * * * * * * * * * ?"

Answer: (I always try to be magnaminous in answering this question, especially if the dentist named is a reputable practitioner; one not likely to be injured much, anyhow, by malicious shafts. If he is one of that class, I let him off easy, by saying, for instance, that he makes a pretty fair filling, which won't come out if you are a little careful about eating taffy, and about using tooth-picks, and about not riding in jolt-wagons. If he is one of the disreputable class; that is to say, if he is a young practitioner and hasn't as large an income as mine, and if he has no assistant to mallet for him, I smile in a peculiar way and refer the questioner to my partner, who never evades a truthful answer; he replies, in effect, that Dr. Blank has much to learn, that he was told recently that the gentlemen in question couldn't tell an alveolar abscess from a case of hernia, and that he uses cheap teeth on his plates. Dr. Prostheticus is not easily embarassed, but I once saw him when he appeared to be somewhat involved. It was when he remarked, in reference to a young practitioner over the way, that a man, after having practiced dentistry for ten years, usually comes to the conclusion that he does not know anything about it. The listener sighed disconsolately and observed in a compassionate tone, that after twenty or thirty years of practice, a dentist must feel rather dejected when he comes to measure the depths of his ignorance. Dr. P. made no reply that I recall, but fell to musing, and in his absent minded state, signed a receipt for twenty-eight dollars upon payment of fifty cents. The cash account didn't balance that evening, and the error was not detected for several days. But to resume our list of questions and answers.

Question: " * * * * * * * * * * * ?"

Answer: " Why they *ought* to last ten, fifteen, or eighty-five years—I won't pretend to say exactly how long. Of course, you should be careful how you use your teeth. It is'nt advisable to use them to loosen knots in a trace-chain, nor to start the rivets in a boiler-plate."

Question: " How about * * * * ?"

Answer: " Oh there is a good deal of bosh about all that. Walnuts and hickorynuts are not meant to be included in the list of injurious substances. Reference is had to cocoanuts and billiard-balls, and so on, which being very hard, are likely to injure the enamel if you try to crack them in that way. At all events, it is certainly better to start a crack in them first with a hammer."

Question: " How much am I to pay you for this, Doctor?"

Answer: [To this question I respond, (after a moment's consideration, to enable me to form a rough estimate of the patient's pile,) fifty dollars, or seven hundred and fifty dollars, [as the Genius of the moment inspires me.]

Question: "How much are you going to gouge me, Doctor?"

Answer: "Oh, say three dollars and ten cents."

Question: "Shall I pay you * * * * * * * * * * next week?"

Answer: "Now, if you please." (I always keep a note of four hundred dollars in bank, maturing within twenty-four hours of any date I choose to assign. This is a very convenient arrangement, and saves a man from the necessity of inventing naughty stories about being in arrears at the dental depot, or at his boarding house. Besides, there's a good deal more style about it.")

There are numerous other questions which I have not time now to enumerate, but which I intend to arrange and classify at an early date, for the benefit of any who feel a curiosity to compare their experience with mine. For these questions, I have an extensive assortment of effective answers, which will be published for the benefit of the profession soon. I am willing to be placed on record as affirming that the mallet-boy of the future will be required to possess intellectual capacity sufficient to enable him to answer any or all of these questions with tact and skill, while his employer is washing his hands, or absents himself from the office to procure a clove. Of course, the propriety of bringing this list of questions to the attention of dental students, is at once suggested. We fill our students up with vast quantities of information on the subjects of anatomy, physiology, chemistry, pathology, etc., but how many of them go forth from their Alma-Mater prepared to wrestle with the complex, double-convoluted, inconsequential queries propounded by the pestiferous beings whose patronage they seek? A man comes into the young practitioner's reception-room at the very moment he is recording an appointment with Miss C., daughter of the wealthiest man in the town. Miss C. has heard that the new dentist has some new patent way of putting in fillings so that they won't come out. Most inopportunely, the new arrival breaks out with: "Doctor, that plug you put in my tooth last month came out the very next day. What is the reason of that?" Our unhappy young practitioner blushes nervously, while Miss C's mother begins to elevate her eyebrows, and glances significantly at her daughter. The next day our y. p. receives a note from Mrs. C. expressing regret that owing to a change in the family's arrangements about going to Long Branch, her daughter wishes to postpone her appointment indefinitely. Several months later, he learns casually, that Miss C's father paid a dentist in New York three hundred dollars for services rendered the young lady.

Now our y. p's Alma-Mater is indirectly responsible for all this. He ought to have been forewarned of the probability of such an emergency's presenting. We could have enlightened the y. p. by informing him that he had the interloper at a disadvantage from the very first. What could have been more adroit than to have smiled incredulously, and to have offered, playfully, to wager a silk hat against a piece of lemon-peel, either, 1stly, that the filling had *not* come out, or, 2ndly, that it was not his filling if it *had* come out, or, 3rdly, that the tooth never had been filled? Meanwhile, the office-boy (of the future,) such a one as we have at *present*, would have been instructed to open the door for Mrs. C. and daughter, and the strategic details of the scheme would have been complete.

One more observation before we close. In the midst of all this disturbance about dental education, let some one, ingenious in such matters, set about investigating how Mons. Hermann, the magician, contrives to tell, without looking, how much money a man has in his pocket-book. For the possession of this secret, the profession would be willing to pay a royalty that would make the stock-holders of the Cummings Dental Vulcanite patent green with envy. It is our private opinion that the one thing really needed to place our profession on an equality with the legal and medical professions, is the means to live in stone-front houses and furnish our wives with seal-skin sacques. As matters are at present, we are in danger of being distanced by the plumbers.

